# Health Committees under the Insurance Bill

**Published:** 1911-07-01

**Authors:** 


### The Hospital

A Journal of

The Medical Sciences and Hospital Administration.

New Series. No. 231, Vol. IX. [No. 1303, Vol. L.] SATURDAY, JULY 1. 1911

### HEALTH COMMITTEES UNDER THE INSURANCE BILL.

One of the most interesting clauses in the National Insurance Bill which is now before Parliament is that dealing with the formation and constitution of local Health Committees to whom, if the Bill becomes law, is to be entrusted the duty of supervising the carrying out of certain parts of the Act. Such committees will be of the greatest service to the State and to the community. Indeed, it is hardly an exaggeration to say that without them the working of the measure will be found almost impracticable, while its clauses will certainly have a stigma of officialdom that the public is hardly likely to tolerate for any length of time. By the formation of such local committees provision is made for the creation of a necessary and highly useful auxiliary to the Commissioners. The application of the Continental Acts, as we have shown in the series of papers now appearing in The Hospital, has been greatly aided by the enlistment of local co-operation and local effort. Under the present Bill, the Health Committee is empowered "to consider generally the needs of the county or county borough with regard to all questions of public health, and to make such reports and recommendations as it may think fit, and further to make provision for the giving of lectures and the publication of information on questions relating to health." The Bill specifically states that the committee may enlist the assistance of the local medical officer of health, and apparently gives fairly wide powers to the body itself. No one who has at heart the public interest with regard to health and hygiene can do otherwise than cordially appreciate the action of the Government in at last making some endeavour to deal with a very serious national problem in a manner that is likely, provided the Health Committees are carefully chosen, to prove of the greatest value to the community.

Section 43 of the Bill defines the conditions pertaining to the appointment of such local Health Committees, and we would earnestly direct the attention of all students of the measure to this clause. As it stands, it is open to wide amendment. One-third of the committee, so the Bill states, is to consist wholly or in part of members of the local sanitary authorities, appointed by the county or borough council; another third is to be elected by the local insurance societies, or if such societies cannot agree upon the appointment, then by the Insurance Commissioners, while the remaining third is to be appointed "by any association of deposit contributors resident in the county or county borough which may have been formed under regulations made for that purpose by the Insurance Commissioners, provided that if no such association has been formed, such one-third shall, subject to the approval of the Insurance Commissioners, be appointed by other members of the committee or in default by the Insurance Commissioners, from amongst, so far as practicable, such deposit contributors." In addition to the members appointed under these three provisos, who are not to exceed eighteen in number, the Commissioners are to appoint another four, of whom two at least shall be duly qualified medical practitioners. From this it appears that the number of members in such a Health Committee would average about twenty, of which three-quarters may, under certain conditions, be nominees of the Commissioners.

The first thing that strikes one in considering such a committee is the size of it. Municipal experience has shown that the best work is done by small committees, composed of men who are earnest disinterested workers, who have no axes to grind and no fads to defend. If the proposed Health Committees are to achieve the amount of success that it is legitimate to expect from them, they will have to be composed of such workers—recruited from the ranks of public-spirited men who are capable and willing to devote their energies to the consideration of vital questions of public health. A large part of the business that comes before such a body will be of a highly technical kind, and it is therefore imperative that among the members there should be local experts who are thoroughly qualified to deal with such subjects as they arise. Provision has been made to enable the committees to obtain the help of the local medical officer of health, and it is apparently taken for granted that the sanitary authorities will be able to deal with certain other aspects of these questions owing to their experience gained through serving on the borough council. But it is worth while bearing in mind that municipal councillors approach health questions from a point of view different from that taken by outside workers. It is true that provision has been made for the inclusion of such outsiders, but it seems to us that the Bill gives too wide powers to the members of the committees themselves to co-opt members. The inclusion of at least two medical men is a safeguard which will be generally appreciated, but those who have had experience of the working of lay bodies of which one or two experts are members will readily agree that there is always a danger that too little significance will be attached to the opinion emanating from experts. The work which the committees will have to deal with under the Bill is of a specially debatable character, and it is desirable that further provision should be made, in the interests of the community, to obviate the possibility of business and other local considerations unduly influencing the committees in their recommendations. For example, the question of proper housing will have to be dealt with, and it is, therefore, necessary that the influence of the jerry-builder and the large contractor, unfortunately so often apparent in municipal recommendations, should be rigorously excluded.

Amendments to the clauses in the Bill relating to the composition of Health Committees are to be tabled when the Bill comes up for discussion, and it behoves the public to follow attentively their tenor and meaning. We have pointed out two weak spots in the provisions as printed—the undue proportion of members assigned to purely lay organisations by the principle of co-option, and the undue size of the committees themselves. Both these can be easily dealt with. For all practical purposes a committee of a dozen members is quite sufficient, and a fourth of these, at least, should be men with expert experience of hygiene, sanitation, public health, engineering, and organisation.

